# Incidence and Outcomes of Neutropenic Enterocolitis Post-stem Cell Transplant for Hematological Malignancies: A Retrospective Study in Australia

**DOI:** 10.7759/cureus.87560

**Published:** 2025-07-08

**Authors:** Sumy Francis

**Affiliations:** 1 Intensive Care Unit, Epworth Freemasons Hospital, East Melbourne, AUS

**Keywords:** autologus stem cell transplant, haematological malignancies, hematological malignancies, intensive care and invasive monitoring, neutropenic colitis, neutropenic infection, neutropenic sepsis

## Abstract

Neutropenic enterocolitis (NE) is a well-known occurrence in patients who receive chemotherapy and stem cell transplantation for hematological malignancies. Nonetheless, there is a lack of Australian research data available on this life-threatening condition. The purpose of this study is to determine the percentage of patients with multiple myeloma and lymphoma who develop NE after autologous stem cell transplant (ASCT) in an Australian setting and to examine the mortality rates in the selected group. Retrospective data were collected from a small pilot group of patients (N = 33) at a private hospital in Melbourne, Australia, who underwent ASCT between February 2023 and December 2024 for multiple myeloma and lymphoma. Patients received either carmustine, etoposide, cytarabine, and melphalan (BEAM); melphalan; or thiotepa, busulfan, and cyclophosphamide (TBC) as conditioning chemotherapy. Patients were then transplanted with peripherally collected CD34+ (positive) cells. Results indicate that 100% of the patient population developed neutropenia, characterized by an absolute neutrophil count (ANC) of 0.0 × 10⁹/L and gastrointestinal (GI) symptoms, including diarrhea. Patients who received the BEAM conditioning regimen developed absolute neutropenia earlier than those who received different conditioning regimens. Twenty (60%) patients developed neutropenic infections confirmed by positive microbiology results. *Escherichia coli (E. coli)*, *Staphylococcus aureus (S. aureus)*, *Clostridioides difficile* (*C. difficile*), and Norovirus were the main causative organisms for neutropenic infections in the study population. Thirty-one (94%) patients were treated with broad-spectrum antibiotics. A positive correlation was found between the onset of absolute neutropenia and the onset of GI symptoms. Ten patients (30.3%) had radiographic evidence confirming NE. The incidence of NE in this study is higher (30.3%) than in previous findings (9%) published in the United States, while the mortality rate remains low (6%). The low mortality rates are likely attributed to close monitoring, early screening, intensive care unit (ICU) management, and the aggressive use of broad-spectrum antibiotics. All patients were managed conservatively without needing surgical intervention. It was also identified that there is a need for standardized criteria for the diagnosis of NE based on clinical presentation, rather than the current guidelines, which include neutropenia with a neutrophil count of <0.5 × 10⁹/L, fever, abdominal pain or diarrhea, and the need for a radiographic evidence of bowel wall thickening >4mm for confirmation of the diagnosis of NE. The reliance on international literature on this topic, combined with the scarcity of Australian-specific studies, underscores the importance of this study and its findings. The low mortality rates found in this study are promising for patients undergoing ASCT for hematological malignancies.

## Introduction

Autologous stem cell transplant (ASCT) refers to the collection of a patient's own hematopoietic progenitor CD34+ stem cells (either from the bloodstream or bone marrow) and their reinfusion back to the patient after chemotherapy for hematological malignancy [1]. ASCT can help reproduce healthy bone marrow cells and replace the damaged cells resulting from chemotherapy. Neutropenic enterocolitis (NE) or typhlitis refers to a gastrointestinal (GI) infection related to neutropenia (decreased neutrophil count in the blood) arising from cytotoxic chemotherapy, which is given as a conditioning regimen before ASCT. NE is characterized by GI tract mucosal injury, microbial invasion, transmural bowel wall inflammation and oedema, which may progress into ulceration, necrosis, bowel perforation, peritonitis, and sepsis. It often presents as fever, abdominal pain, diarrhea, and abdominal distension [2,3]. The caecum and terminal ileum are often affected due to the presence of lymphoid follicles, Peyer’s patches, and their active involvement in immune defence. Infections can be polymicrobial and may involve a combination of Gram-positive cocci, Gram-negative rods, anaerobes, and fungi. While there are no standardized criteria for the diagnosis of NE, the most commonly used criteria in clinical settings include fever >38°C, abdominal pain or diarrhea, neutropenia with an absolute neutrophil count (ANC) <0.5 × 10^9^/L, and bowel wall thickening >4mm on radiological investigation [4]. More clinical guidelines are required to aid the diagnosis of NE, rather than relying solely on radiographic evidence for confirmation of NE.

Chemotherapy-induced neutropenia and associated NE are common complications in immunocompromised patients, carrying a high mortality rate [5]. Nevertheless, a literature search over the last eight years has yielded only a few international studies on this topic, most of which were published as case series or meta-analyses. There is a scarcity of Australian-specific data providing insight into NE's national incidence and mortality rates. The limited Australian research published on this topic was almost 30 years ago [6,7], and the studies reported mortality rates as high as 50% for those who were diagnosed with NE. Some studies in the past referred to NE as "abdominal surgical disease," and there were a greater number of laparotomies performed [6,7]. The findings of this study will provide insights into the most recent data on NE in an Australian context, given the significant differences between the Australian healthcare system and those of other countries mentioned in the international literature.

A study published in the United States in 2010 estimated that the incidence of NE following stem cell transplant over five years was approximately 9%, with a mortality rate of 12.5% [8]. Six patients (18%) in this study [8] required admission to the intensive care unit (ICU); however, most patients required only conservative management, which included bowel rest, intravenous fluids, and parenteral broad-spectrum antibiotics, without the need for surgical intervention. A similar conservative management strategy was reported by Belmoufid et al. [5] in their case series report conducted in Morocco. Brunel et al. [9] support this trend of declining mortality rates in their study, attributing it to advanced clinical screening, prompt ICU transfer, close monitoring, and effective management of septic shock. Their study reported a mortality rate of 6.5% [9].

The primary objective of this study is to understand the incidence of NE in patients who received conditioning chemotherapy and subsequent ASCT for hematological malignancies like multiple myeloma and lymphoma. The secondary objective examines the mortality rates following the development of NE. It is hypothesized that the incidence in Australia will be similar to that observed in the study by Jimenez et al. [8] in the United States, characterized by low incidence and mortality rates, with most cases being managed conservatively without requiring surgical intervention.

## Materials and methods

Retrospective data were collected from 33 adult patients at a private hospital in Melbourne, Australia, who underwent ASCT between February 2023 and December 2024 for hematological malignancies. The study site is a private hospital predominantly treating hematology and oncology patients. The site is also the first private hospital in Victoria, Australia, to conduct ASCT. For this reason, the study will be considered a pilot study with a small sample size. Ethical approval was received from the participating institution (EH2024-1157).

Method

Medical records of 33 adult patients who underwent ASCT at the study site between February 2023 and December 2024 for multiple myeloma and lymphoma were reviewed. The onset of neutropenia, fever, GI symptoms, escalation of care, antibiotic use, and granulocyte colony-stimulating factor (G-CSF) use, as well as microbiology and radiology findings, were examined from the day of ASCT. The principal investigator abstracted all data from the archived hospital medical records. The inclusion criteria considered all subjects, both males and females over the age of 18, who were admitted to the study site between February 2023 and December 2024 for elective ASCT for multiple myeloma or lymphoma. The stage of their disease or the type of conditioning treatment received before ASCT was not a limiting factor. The pediatric population under the age of 18 years was excluded from the study. Ten patients (30.3%) out of 33 had a confirmatory diagnosis of NE following a computed tomography (CT) scan, as reported by the radiologist. In contrast, the other 23 patients (69.6%) received a presumptive diagnosis of NE based on their clinical symptoms, such as fever, abdominal pain or diarrhea, and neutropenia (count <0.5 × 10^9^/L), but without radiographic evidence. The commonly used criteria for the diagnosis of NE, i.e., neutropenia with a count <0.5 × 10⁹/L, fever, abdominal pain or diarrhea, and radiographic evidence of bowel wall thickening >4mm, were used as a benchmark for determining the incidence of NE in the sample population. The median duration of GI symptom onset was calculated from the day of ASCT, which was designated as day zero. Survival or mortality rates were also noted at the end of the relevant hospital admission episode for autologous stem cell transplant.

Other variables explored in this study were the incidence of neutropenic infections other than NE, type of causative organisms identified, type of antibiotics used, median duration to commencement of antibiotics, median duration to the development of absolute neutropenia (ANC of 0.0 × 10⁹/L), median duration to the development of GI symptoms post-ASCT, the average duration of GI symptoms, results of CT scan to confirm the diagnosis of NE, the median duration of ICU stay, the median duration of hospital stay, rate of the use of G-CSF for the support of neutrophil recovery, the median duration of G-CSF use, the median duration to neutrophil engraftment and overall survival or outcome post-ASCT. The VassarStats statistical computation website was used for statistical analysis.

Out of the 33 patients, 12 were females (36.4%), and 21 (63.6%) were males. The mean age of the study population was 62 years (range 26-76). Multiple myeloma was the most common diagnosis (63%) treated. Thirty-two patients (96.9%) had a diagnosis of either multiple myeloma or lymphoma, and one patient had amyloidosis, with a history of multiple myeloma in remission. The subtypes of lymphoma treated using ASCT included follicular lymphoma, non-Hodgkin’s lymphoma, Hodgkin’s lymphoma, diffuse large B-cell lymphoma, peripheral T-cell lymphoma, mantle cell lymphoma, and primary CNS lymphoma. Patients received conditioning chemotherapy using carmustine, etoposide, cytarabine, and melphalan (BEAM), thiotepa, busulfan, and cyclophosphamide (TBC) or melphalan. They were then transplanted with CD34+ stem cells collected from peripheral blood. Table 1 shows the descriptive statistics of the sample population.

**Table 1:** Descriptive statistics of patient characteristics (N = 33)

Patient characteristic	Value	Percentage
Total sample size	33	
Diagnosis		
Multiple myeloma	21	63.6%
Follicular lymphoma	1	3.03%
Non-Hodgkin's lymphoma	1	3.03%
Hodgkin's lymphoma	1	3.03%
Diffuse large B-cell lymphoma	2	6.06%
Peripheral T cell lymphoma	1	3.03%
Mantle cell lymphoma	3	9.09%
Amyloidosis	1	3.03%
Primary CNS lymphoma	2	6.06%
Sex		
Male (M)	21	63.6%
Female (F)	12	36.4%
Conditioning treatment received		
Melphalan (140, 180, & 200)	22	66.66%
TBC	2	6.06%
BEAM	9	27.27%
Mean age (range)	62 (26-76) (SD = 11.65)	

## Results

**Figure 1:**
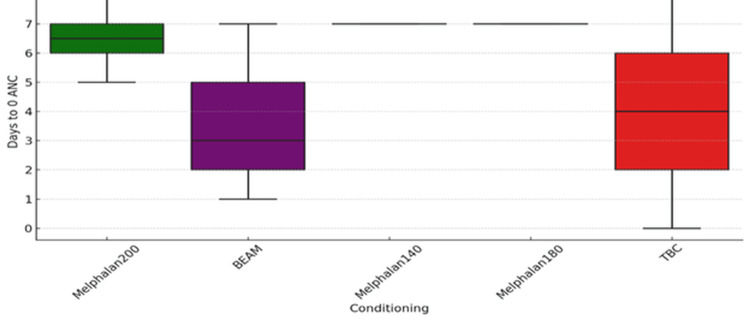
Days to absolute neutropenia commencement vs type of conditioning regimen used (p = 0.001)

All participants in the study developed absolute neutropenia with an ANC of 0.0 × 10⁹/L on the days following conditioning chemotherapy and ASCT. The median number of days to the development of absolute neutropenia was six (range, 0-8). Patients who received the BEAM conditioning regimen developed absolute neutropenia earlier (mean = 3.5 days, SD = 1.8) than the patients who received other types of conditioning regimens. The mean duration of the development of absolute neutropenia was four days for TBC (SD = 5.6) and 6.5 days for melphalan 200 (SD = 0.82). The wide standard deviation for TBC is attributed to the small sample size. The boxplot below (Figure 1) illustrates the differences in the development of absolute neutropenia among groups that received different conditioning regimens.

Analysis of variance (ANOVA) statistical analysis and comparison between the different groups (melphalan, TBC and BEAM) showed an F-value of 6.01 and a p-value of 0.001. Tukey's post-hoc analysis identified the only significant groups for comparison as those between BEAM and melphalan 200 (p = 0.000), with the BEAM regimen being associated with an earlier ANC nadir. This indicates that the results are statistically significant, and the patients who received the BEAM conditioning regimen developed absolute neutropenia earlier than those in other groups.

Ten patients out of 33 (30.3%) had either enteritis, colitis or enterocolitis confirmed using a CT scan, ANC of 0.0 × 10⁹/L, abdominal pain and/or diarrhoea, and fever on at least two occasions, consistent with the diagnosis of NE. A total of 100% of patients developed diarrhoea, ranging from mild (three days) to prolonged diarrhoea of 60 days or more; however, not all patients had febrile episodes or radiographical confirmation of enterocolitis or typhlitis. Patients who did not have radiographic evidence (due to physician choice) were excluded from the NE count. Six patients (18%) required ICU admission, and two patients died in the ICU, bringing the overall mortality rate to 6%. Five (83.3%) out of the six patients who were admitted to the ICU had typhlitis with involvement of the ileocaecal valve. ICU admission was determined by clinical parameters, such as fever, tachycardia and persistent low systolic blood pressure (<90 mmHg) requiring vasopressors or inotropes for haemodynamic support.

The median number of days to develop diarrhoea post-ASCT was four days (range, 0-9), and the median duration of GI symptoms was eight days, with a broad range of 3-57 days. Patients with severe bouts of diarrhoea (greater than four watery bowel actions per day) also had cardiac rhythm disturbances or rapid atrial fibrillation, requiring significant electrolyte replacement and/or admission to the ICU for septic shock, ionotropic support and renal replacement therapy.

**Figure 2:**
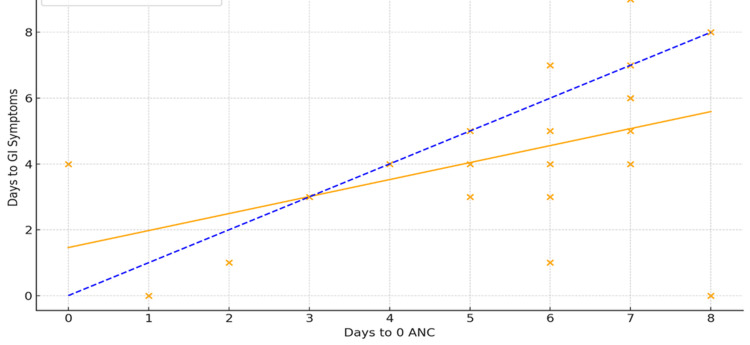
Graph showing a correlation between the onset of neutropenia (number of days to ANC count of 0.0 × 10⁹/L) and the onset of GI symptoms (p = 0.002) ANC: absolute neutrophil count; GI: gastrointestinal

The Pearson correlation analysis evaluated the relationship between absolute neutropenia (when ANC reached zero) and the onset of GI symptoms in patients post-ASCT. The analysis revealed a moderate positive correlation (r = 0.51), which was statistically significant (p = 0.002) (Figure 2, orange regression line). A total of 15.15% of the population demonstrated a linear correlation, as indicated by the blue dotted line in Figure 2. This shows that the onset of neutropenia and GI symptoms positively correlates (Figure 2).

Although a moderate positive correlation was observed between absolute neutropenia and the onset of GI symptoms, the duration of GI symptoms varied. The Pearson correlation analysis between the timing of the onset of GI symptoms versus the duration of GI symptoms showed an inverse relationship between the two variables. The Pearson correlation coefficient was -0.20 (p = 0.197). In essence, the timing of GI symptom onset did not correlate with symptom duration.

**Figure 3:**
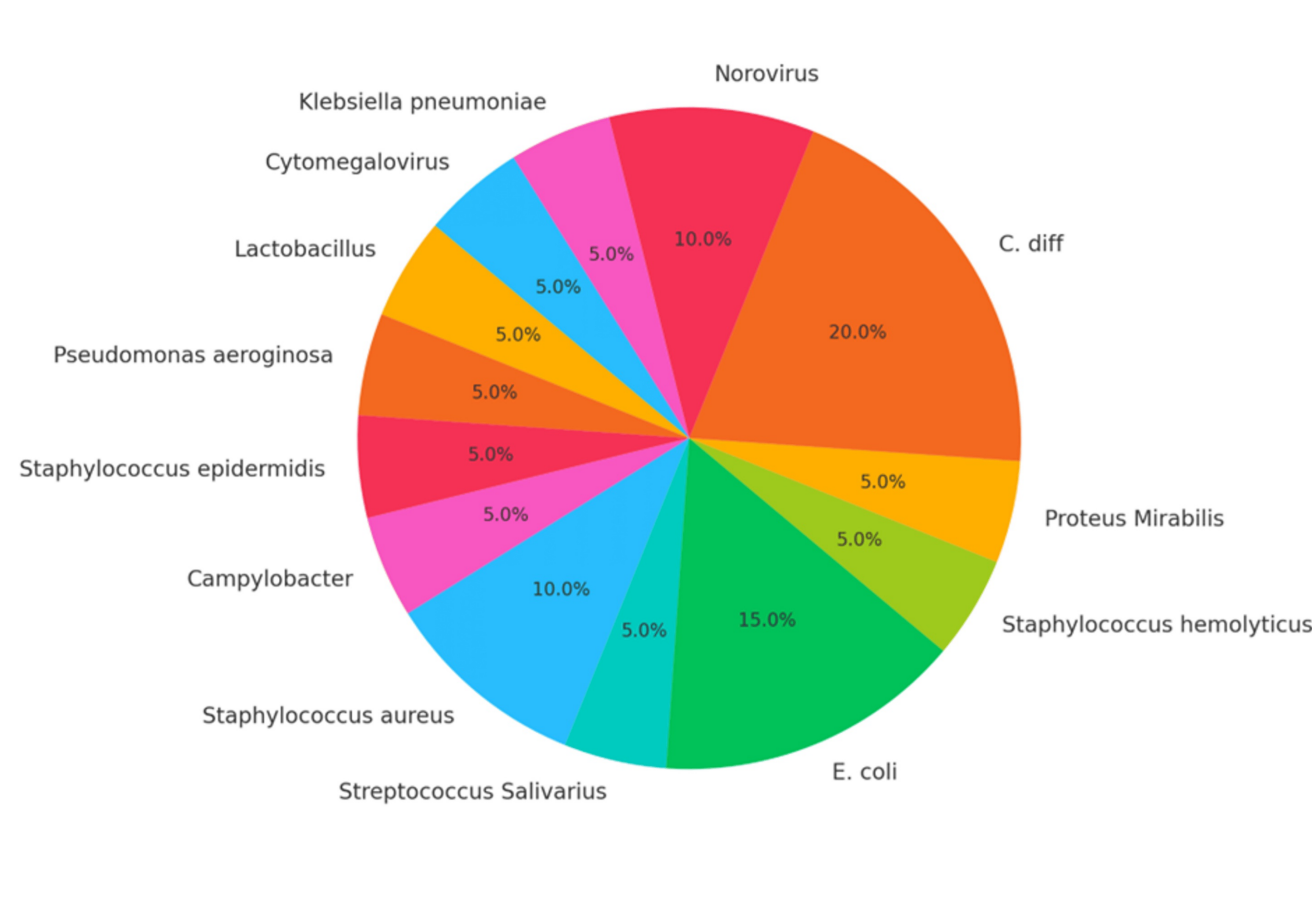
Type of neutropenic infections that occurred in the study population

Neutropenic infections occurred in 20 patients (60.6%). Those patients had positive microbiology results, either from blood, urine or faecal culture. Figure 3 shows the different types of microorganisms identified in the sample population. The most frequently appearing microorganisms were *Escherichia coli* (*E. coli*), *Staphylococcus aureus (S. aureus)*, *Clostridioides difficile* (*C. difficile*), and Norovirus (Figure 3). One patient had cytomegalovirus (CMV) colitis confirmed by colonoscopy and serology, in addition to having *E. coli *bacteremia. This patient died in the ICU. The second patient who succumbed to death in the ICU had *S. aureus* and *C. difficile* infections. Both patients presented with early onset and severe bouts of diarrhoea.

Perianal cellulitis and abscess were uncommon neutropenic infections which required incision and drainage in a young patient. The median duration of a positive microbiology result was six days, aligning with the median of six days for the development of absolute neutropenia.

**Figure 4:**
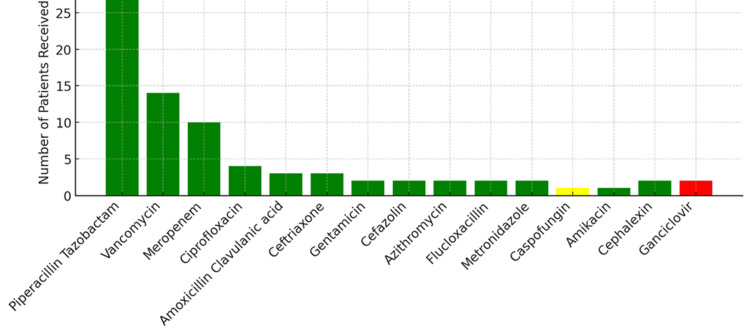
Type of antibiotics, antifungals, and antivirals used in neutropenic infections ^+^Prophylactic use of antimicrobials, antifungals and antivirals is not included in the above chart

Thirty-one patients (94%) were treated with intravenous (IV) broad-spectrum antibiotics. Piperacillin-tazobactam was the most used broad-spectrum antibiotic. All patients received prophylactic oral antiviral valacyclovir and trimethoprim-sulfamethoxazole for prophylaxis of pneumocystis pneumonia. Most patients also received the oral antifungal agent fluconazole, in addition to valacyclovir and trimethoprim-sulfamethoxazole. Additional IV antibiotics were administered when patients received positive blood, faecal or urine culture results (Figure 4). Oral antibiotics used in de-escalation include cephalexin, ciprofloxacin, amoxicillin-clavulanic acid and vancomycin for the treatment of *C. difficile* infections. The median duration to a positive blood culture result and the commencement of antibiotics was 5.5 days. The study confirms that the use of broad-spectrum antibiotics during the neutropenic phase remains high, regardless of the microbiology results.

Twenty-one (64%) patients received total parenteral nutrition (TPN) via peripherally inserted central catheter (PICC), while they were kept on bowel rest. Six patients (18%) required ICU admission for life-threatening septic shock, ionotropic support and multi-organ failure. Five patients (15.15%) required renal replacement therapy for acidosis correction and toxin and fluid removal. Two patients died (6%) in the ICU. The length of ICU stay ranged from eight to 56 days, with a median duration of 16 days. Three (9%) patients required rehabilitation at home or a rehabilitation facility upon discharge, and 28 (85%) were discharged home. The median length of hospital stay was 20 days. Patients discharged to the rehabilitation facility had low to moderate Karnofsky scores prior to ASCT. The two patients who died in the ICU had a perfect pre-treatment Karnofsky score of 100%, which highlights the fatality of neutropenic infections.

All patients were administered subcutaneous G-CSF (94% short-acting G-CSF, 6% long-acting G-CSF*) to aid faster neutrophil recovery. The median duration of G-CSF administration was seven days. Neutrophil engraftment occurred on a median of 11 days (range 8-13). Long-acting G-CSF was administered weekly and short-acting G-CSF was administered daily.

**Figure 5:**
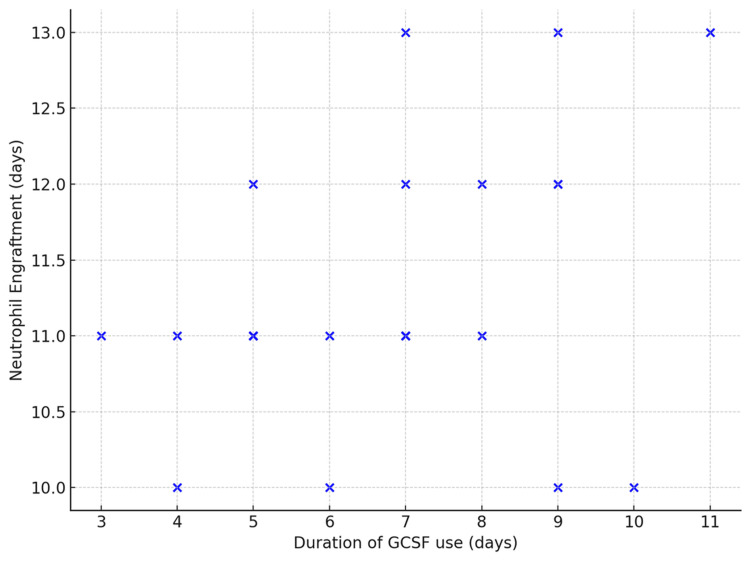
Scatterplot showing the relationship between the duration of short-acting G-CSF use and neutrophil engraftment. Pearson's correlation coefficient (r) = 0.29, p-value = 0.117 G-CSF: granulocyte colony-stimulating factor Long-acting G-CSF administration was not included in the graph to prevent outliers

The scatterplot in Figure 5 analyzes the relationship between the duration of G-CSF use and neutrophil engraftment. The high p-value indicates that the duration of G-CSF use does not significantly impact neutrophil engraftment time. Longer duration of G-CSF use was not associated with earlier neutrophil recovery. Two patients in this study received long-acting G-CSF; they were not included in the scatterplot below.

## Discussion

This single-center, retrospective study was conducted on a small pilot group of patients who underwent autologous stem cell transplants for multiple myeloma and lymphoma. All patients developed absolute neutropenia (ANC of 0.0 × 10⁹/L) and diarrhoea ranging from mild to severe in duration. Patients who received BEAM conditioning were noted to have an early onset of neutropenia, which validates the studies by Gil et al. [1]. It is worth noting that the delayed onset of GI symptoms was not associated with the duration of GI symptoms experienced.

Treatment of NE and the use of G-CSF

Neutropenic patients were treated promptly with broad-spectrum antibiotics without waiting for a positive microbiology result. Thirty-one (94%) patients received a broad-spectrum antibiotic, piperacillin-tazobactam, for seven days. The duration of antibiotic therapy and the appropriate course of treatment for neutropenic infections are beyond the scope of this study. Pravato et al. [10] compared time to clinical improvement in patients who received both short-term and long-term antibiotic therapy for neutropenic infections and found them to be similar. Further studies are needed to gain a deeper understanding of Australian statistics. All patients in our study received G-CSF; however, the use of G-CSF remains a topic of controversy. Our study showed that a longer duration of G-CSF use was not correlated with earlier neutrophil engraftment. Zafrani and Azoulay [11] suggest that G-CSF does not improve clinical outcomes and that granulocyte infusions risk CMV transmission in neutropenic patients.

Ten (30.3%) out of 33 patients had confirmation of bowel irritation and inflammation using a CT scan. There was a predilection for inflammation of the terminal ileum and caecum for reasons mentioned before; however, other areas of the bowel were also involved. The wide range of findings on CT scan reports for these 10 patients includes a combination of enteritis, ileitis, typhlitis, segmental colitis, diffuse colitis, proctitis and pancolitis. Five of the six patients admitted to the ICU had evidence of ileocecal valve inflammation on CT scan. One patient died in the ICU on day 1, and a CT scan was not obtained earlier for that patient. Surgical consultations were sought, and the decision was made to manage conservatively. A recent systematic review by Nematolahi et al. [12] concluded that the involvement of the ileocecal valve was significantly at odds with increased mortality rates, including sepsis and multi-organ failure.

Radiological imaging for the diagnosis of NE

It is worth noting that all patients in this study developed diarrhoea during the neutropenic phase, but not all experienced febrile episodes or underwent CT scans to confirm the diagnosis of NE. All patients who developed diarrhoea (but did not have CT scan evidence) were given a presumptive diagnosis of neutropenic colitis or typhlitis based on their clinical presentation, and they were all treated with broad-spectrum antibiotics, like those who had radiographic confirmation. This warrants a review of the widely used current criteria for diagnosing NE, which include the triad of neutropenia (neutrophil count <0.5 × 10⁹/L), fever, abdominal pain or diarrhoea, and bowel wall thickness >4 mm on CT. A revised diagnostic criterion, potentially eliminating the need for a confirmatory radiograph, would be beneficial in initiating prompt treatment in vulnerable immunocompromised patients.

We conclude that the incidence of NE (30.3%) in this study is higher than anticipated, possibly due to close monitoring, reporting and prompt screening. This contrasts with the 9% hypothesis reported in the Jimenez et al. [8] study. This estimate of incidence only accounts for CT-scan-confirmed cases of NE. Our study shows that 100% of patients developed diarrhoeal illness, and 94% of the population were treated with broad-spectrum IV antibiotics. The incidence of ICU admission (18%) was comparable to that in previous studies published in the United States [8], and overall mortality remained low (6%), likely due to early screening, prompt management and timely ICU admissions. The low mortality rate found in this study (6%) is aligned with the 6.5% mortality rate reported by Brunel et al. [9] in their internationally published research, and in contrast to the high mortality rates (30-50%) reported in the study by Ullery et al. [2]. The declining trend in mortality rates over the years points to advancements in medical and nursing care. Patients undergoing autologous stem cell transplant should be educated on NE and the precautions to be taken during the neutropenic phase.

Limitations

The study has several limitations, including a small sample size and a single-center design. It was limited to patients who had ASCT, primarily for multiple myeloma and lymphoma. The study was conducted in an Australian fee-paying private hospital, where social determinants might impact patients' improved health and clinical outcomes. The findings of our study may not correlate with the patient presentations in the country's larger tertiary public hospitals. More large-scale studies specific to Australian settings are required for better statistical analysis. The results cannot be extended to the paediatric population and other adult patients who had allogeneic stem cell transplants for hematological malignancies.

## Conclusions

In conclusion, the incidence of neutropenic enterocolitis following autologous stem cell transplant is high in this study, possibly due to close monitoring, reporting, and early screening. The small sample size could also be potentially contributing to the higher rate of incidence. The occurrence rate excludes patients who had no radiological confirmation of NE, and the actual rates may be even higher than those reported in this study. Nevertheless, those patients who had no CT scan evidence of NE presented with similar clinical symptoms (fever, abdominal pain, diarrhea, and absolute neutropenia) to those who had a diagnosis of NE, and most of these patients were treated similarly with broad-spectrum antibiotics. Despite the high incidence, mortality rates remain low, likely due to prompt management, ICU care, and the use of IV broad-spectrum antibiotics. There is a need for a standardized diagnostic criterion for diagnosing NE based on the clinical symptoms mentioned above. Early empirical treatment of NE, even in the absence of imaging, could potentially avoid life-threatening complications like sepsis and multi-organ failure. Reliance on radiographic evidence for a confirmation of diagnosis could delay treatment and may prove fatal. The study also confirms that NE can be managed conservatively with broad-spectrum antibiotics, bowel rest, total parenteral nutrition, and ICU admission for sepsis or life-threatening complications. None of the patients in this study required surgical intervention. More Australian multicenter studies are needed on this topic for better statistical analysis.
